# An Access Control System Based on Blockchain with Zero-Knowledge Rollups in High-Traffic IoT Environments

**DOI:** 10.3390/s23073443

**Published:** 2023-03-24

**Authors:** Xin Lin, Yuanyuan Zhang, Changhai Huang, Bin Xing, Liangyin Chen, Dasha Hu, Yanru Chen

**Affiliations:** 1 School of Computer Science, Sichuan University, Chengdu 610065, China; 2 Sichuan GreatWall Computer System Co., Ltd., Luzhou 646000, China; 3 Chongqing Innovation Center of Industrial Big-Data Co., Ltd., Chongqing 400707, China; 4 National Engineering Laboratory for Industrial Big-Data Application Technology, Beijing 100040, China; 5 Institute for Industrial Internet Research, Sichuan University, Chengdu 610065, China

**Keywords:** blockchain, access control, zero-knowledge proof, zero-knowledge rollups

## Abstract

The access control (AC) system in an IoT (Internet of Things) context ensures that only authorized entities have access to specific devices and that the authorization procedure is based on pre-established rules. Recently, blockchain-based AC systems have gained attention within research as a potential solution to the single point of failure issue that centralized architectures may bring. Moreover, zero-knowledge proof (ZKP) technology is included in blockchain-based AC systems to address the issue of sensitive data leaking. However, current solutions have two problems: (1) systems built by these works are not adaptive to high-traffic IoT environments because of low transactions per second (TPS) and high latency; (2) these works cannot fully guarantee that all user behaviors are honest. In this work, we propose a blockchain-based AC system with zero-knowledge rollups to address the aforementioned issues. Our proposed system implements zero-knowledge rollups (ZK-rollups) of access control, where different AC authorization requests can be grouped into the same batch to generate a uniform ZKP, which is designed specifically to guarantee that participants can be trusted. In low-traffic environments, sufficient experiments show that the proposed system has the least AC authorization time cost compared to existing works. In high-traffic environments, we further prove that based on the ZK-rollups optimization, the proposed system can reduce the authorization time overhead by 86%. Furthermore, the security analysis is presented to show the system’s ability to prevent malicious behaviors.

## 1. Introduction

The Internet of Things (IoT) connects parts of machines, devices, users, and intelligent systems on a massive scale [1,2]. It is anticipated that by 2022, there will be 50 billion connected IoT devices [3,4], and this enormous expansion demonstrates that IoT systems will be extensively used in an increasing number of fields (e.g., smart transportation, smart healthcare, smart city, smart grid, etc.) and will significantly enhance the user experience in different scenarios. Although the application of IoT systems has made our lives easier, it also results in many security and privacy issues, among which the access control (AC) [5,6] issue is particularly prominent.

The access control issue can be described as being based on a security mechanism to guarantee that only authorized entities access specific IoT devices. Common access control methods include mandatory access control (MAC) [7], role-based access control (RBAC) [8], attributed-based access control (ABAC) [9] and capability-based access control (CapBAC) [10]. AC systems include traditional centralized AC systems and distributed AC systems [11]. Traditional AC requires a central entity for decision-making, which is unsuitable for IoT environments with a wide distribution of IoT devices and complicated networks, since it will likely suffer from major single points of failure, and the blockchain-based AC system is thought to be the best-distributed architecture [12,13,14,15,16,17,18]. In advance, to address the protection of user privacy, the blockchain-based AC system with zero-knowledge proof (ZKP) [19,20,21] has recently gained popularity as a study area.

However, existing blockchain-based AC systems with ZKP [19,20,21] focus on solving how to keep sensitive data from being exposed, but ignore two aspects of the problems. On the one hand, systems built by these works suffer from low transactions per second (TPS) and high latency, and do not take full advantage of on-chain ZK verification to mitigate on-chain computation overhead, since they are not adaptive to high-traffic IoT environments with many users and devices. On the other hand, these works cannot fully guarantee that all user behaviors are honest; a malicious user can bypass pre-defined rules to attack systems, such that an IoT device owner has the opportunity to grant the access token without a target request.

This work proposes a blockchain-based ABAC system with zero-knowledge rollups to solve the above problems. We encode the authorization matching of access attributes and policies with a specialized Merkle proof and a hash puzzle into arithmetic circuits, then generate zero-knowledge proofs through the  zkSNARK framework [22,23]. As a result, the smart contract can obtain the authorization result through zero-knowledge verification, which can ensure the honesty of all participants involved and the confidentiality of sensitive data. Based on the concept of ZK-rollups [24], the proposed system unifies numerous access control authorization requests into a zero-knowledge proof and sends it to the smart contract for batch verification, which lowers the on-chain computation overhead and time cost required for authorization in high-traffic IoT environments.

The main contributions of this paper are as follows:

(1) We propose the ZK-rollups of the access control authorization process, where the authorization matching and access token grant processes from different access control requests can be grouped into the same batch of transactions to generate the zero-knowledge proof uniformly.
(2) We propose a specialized zero-knowledge proof, to ensure that participants involved in the authorization can be honest and that the sensitive data in the authorization matching process will not be leaked. Furthermore, we develop a practical system that supports zero-knowledge access control authorization and demonstrates the complete arithmetic circuit generation algorithms and the protocol implementations.
(3) We design sufficient experiments to prove that the proposed system is suitable for high-traffic IoT environments and provide security analysis to verify the system’s ability to guarantee honesty.

The structure of this work is as follows: Section 2 introduces the related work, Section 3 introduces an overview of the system, Section 4 introduces generation algorithms of the arithmetic circuits required for the zero-knowledge proof and the protocol required to implement the proposed system, Section 5 introduces the implementation details of the proposed system and the experimental results, Section 6 introduces the security analysis which shows the ability of the proposed system to ensure the honesty of participants, and Section 7 concludes this paper.

## 2. Related Work

### 2.1. Attribute-Based Access Control

The term “attribute-based access control” (ABAC) [25], also known as “policy-based access control”, refers to an access control paradigm in which the decision as to whether a subject is authorized to perform some operations by evaluating a set of attributes. ABAC is a technique for enforcing access control policies that are highly adaptable and can be adjusted using a variety of attributes, making it appropriate for usage in distributed or quickly changing contexts. ABAC policy rules are generated as boolean functions of the subject’s attributes, the object’s attributes, and the environmental attributes. An access control authorization is performed by both PEP (Policy Enforcement Point) and PDP (Policy Decision Point) [26], PEP is where authorization is enforced, and PDP is where authorization is made. The ABAC authorization process can be described as follows: the subject submits its attributes to PEP, PEP submits the subject’s attributes to PDP, PDP combines object, subject, and environmental attributes into access attributes to match with the pre-defined policy, PDP returns the matching result to PEP, and then PEP informs the subject. The matching process of access attributes and policies is called authorization matching.

### 2.2. Blockchain Technology

Satoshi Nakamoto initially suggested the blockchain in 2009 [27]. The first widely used peer-to-peer trustless electronic currency was called Bitcoin. Since then, a variety of new digital currencies (known as cryptocurrencies) have been developed using a similar framework. In the meanwhile, other blockchain-based apps have been created throughout the years to execute scenarios other than cryptocurrency. One of these is the IoT scenario.

Directly implemented in blockchain networks, smart contracts [28] are computer protocols that simplify, verify, or enforce contract negotiation. Without requiring human interaction, smart contracts offer the ability to directly track and enforce intricate agreements between parties.

### 2.3. zkSNARK and Zero-Knowledge Rollups

A method known as the zero-knowledge proof (ZKP) allows one party (the prover) to convince another party (the verifier) that a specific statement is true, while the prover refrains from revealing any information other than the fact that the statement is indeed true [29,30]. Zero-knowledge Succinct Non-interactive Argument of Knowledge, or zkSNARK [22,23], is a popular form of non-interactive zero-knowledge proof today. Using zkSNARK, the prover and verifier do not need to directly communicate, and the generated proof is brief and simple to verify. We will follow up with an informal definition of zkSNARK, see Bitansky et al. [23] for a complete introduction. Given an arithmetic circuit *C* that encodes the proposition mathematically, a zkSNARK is defined as a triple of algorithms (KeyGen,Prove,Verify) where:

(1) $$\left(pk,vk\right)\leftarrow KeyGen\left(1^{\lambda },C\right)$$

(2) π←Prove(pk,x,w)

(3) $$\left\{0,1\right\}\leftarrow Verify\left(vk,\pi ,x\right)$$

- KeyGen: Input a security parameter λ and an arithmetic circuit *C*, then use the key generator function KeyGen to probabilistically sample a proving key pk and verification key vk. Both keys can be exposed as public parameters and can be used multiple times.
- Prove: Given a proof key pk, an input message *x*, and a witness *w*, Prove outputs a non-interactive proof π, which proves the correctness of the circuit *C*. In the zkSNARK framework, the arithmetic circuits *C* are first transformed into the rank 1 constraint system (R1CS) ccs, which in turn generates the *x* and *w* required by Prove.
- Verify: Given verification key vk, the proof π, and input *x*, Verify outputs 1 if the verification is correct, otherwise 0 if it is false.

Because of its low throughput, or low TPS (transactions per second), blockchain technology has a variety of application-related issues. Layer 2 is suggested as a method to boost the TPS and enhance the scalability of the blockchain [31], and the rollup [24] is one of its implementations. At the moment, Ethereum [32] has designated the rollup as one of the major characteristics to be implemented in the upcoming phase. The rollup can be implemented by constructing a corresponding Merkle proof for a transaction to prove its correctness so that it can be correctly submitted to the blockchain and advance the state transfer of the blockchain. In this way, rollups remove the need for execution directly on the blockchain: only the on-chain verification is performed, thus the computational pressure is shifted off the chain. One of the on-chain verifiable computing implementations is the zero-knowledge rollup using the zkSNARK framework.

### 2.4. Blockchain-Based AC Systems with ZKP

Considering the lightweight and low-power nature of most IoT devices, processes involved in the access control process, such as policy storage, cryptographic operations, or additional network access, need to be performed by a central entity as an intermediary on behalf of the device, which introduces additional security risks, such as single point of failure or data tampering. Therefore, many scholars introduced blockchain technology as a distributed and trusted entity involved in access controls [33]. In advance, to solve the problem of exposing sensitive data in the blockchain, such that the blockchain becomes vulnerable [34,35,36], many scholars introduced zero-knowledge (ZKP) into blockchain-based AC systems.

The works that are identical to ours include Qinan Li et al. [19], Hu et al. [20] and Maesa et al. [21]. Qinan Li et al. [19] presented a ZK access control protocol, this work was among the first to apply ZKP to the field of AC, but it is limited to the request phase and does not consider the risk of replay attacks. Hu et al. [20] proposed a system to exchange tokens with ZKP of access rights to make IoT devices granted anonymously, it maintains confidentiality on the identities of users. However, this work maintains a Merkle tree with a height of 32 for all tokens, and such a high tree will result in an unacceptable Merkle proof generation time overhead and leaf node storage overhead. Maesa et al. [21] expanded the XACML standard for ABAC systems with the novel concept of private attributes, then built a system that leverages smart contracts and zero-knowledge proofs to allow for transparent policies evaluation without disclosing the value of such sensible attributes. However, this work is not suitable for high-traffic IoT environments. On the one hand, the work is performed on Ethereum to build the system, which provides low TPS and high transaction latency; on the other hand, the much higher-than-average gas consumption of authorization matching means that the system has high computational and storage costs. Moreover, this work does not guarantee the honesty of all participants, and the malicious user has the opportunity to complete the access grant without the existence of the corresponding authorization request.

In summary, identical type of work exists two kinds of problems. On the one hand, these systems suffer from low TPS and high latency, which are not suitable for high-traffic IoT environments; on the other hand, they cannot fully guarantee the honesty of participating users, and malicious users have the opportunity to bypass pre-defined rules to attack systems. Considering the above two issues, we design a special ZKP to guarantee the honesty of users and optimize the blockchain-based AC system based on the ZK-rollup concept to improve the execution efficiency of access authorization, ensuring the stable performance of the system in high-traffic IoT environments. We also design reliable comparison experiments to demonstrate that the proposed system has the best access authorization performance in low-traffic environments and further demonstrate its advantages in high-traffic environments.

## 3. System Overview

In this section, both the participant entities of the system and a sample access control process are presented.

### 3.1. Participant Entities

**Figure 1:**
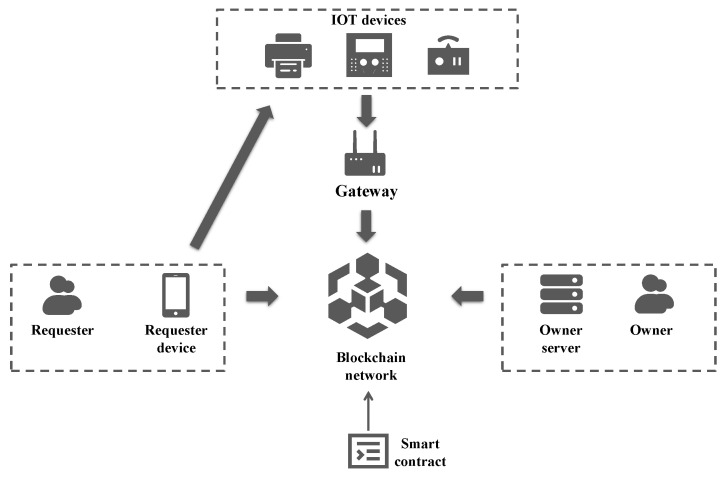
System Overview.

As shown in Figure 1, there are eight participant entities in this system.

- Requester

The requester as an object, who asks for access control privileges for the IoT device as a subject, is the one who starts the access control process. The requester logs into the blockchain network using their device while maintaining anonymity. Other than the cryptographic public key required to encrypt sensitive data and the signature public key used to authenticate calls to smart contracts, it does not reveal redundant information to other network users.

- Requester Device

The requester device acts as an intermediary between the requester and the blockchain network. Through its device, the requester starts the authorization process for access control to the blockchain network and carries requester attributes to facilitate further authorization. If the access control is valid, the requester device will obtain the token it needs from the blockchain network to connect to the IoT device.

- Owner

The owner is the holder of an IoT device in the system, marking that an IoT device belongs to that owner. Similar to a requester, the owner also maintains anonymity throughout the blockchain network. The owner establishes a connection with the blockchain network through its server and performs two duties: (1) the owner needs to register the IoT device with the blockchain network so that the requester can discover the IoT device and initiate access control; (2) the owner can discover from the server that the requester needs to apply for access control to a certain IoT device held by the owner, and store the policies required to authorize requester access control legitimacy in the server.

- Owner Server

The owner server, which holds the owner’s specified policies, serves as a conduit for communication between the owner and the blockchain network. The owner server takes part in the network as a peer node as well. The server must ascertain whether the access attributes satisfy the previously established policies when it obtains authorization from the blockchain network, and create a zero-knowledge proof for a batch of authorization requests to indicate the authorization results (success or failure). The owner server also creates the access token needed to connect to the IoT device.

- IoT Devices

IoT devices are entities that requesters can access. The gateway acts as the intermediary between the IoT device and the blockchain network because the majority of IoT devices have constrained storage and computing capabilities. A requester uses a specific IP address to connect to an IoT device and is equipped with an access token. The IoT device then verifies the token’s legitimacy with the smart contract via the gateway, and if the token is legitimate, the requester’s access is verified.

- Gateway

The gateway is the agent that establishes the connection between IoT devices and the blockchain network. It is responsible for communicating with IoT devices using dedicated structured protocols (e.g., mqtt [37], modbus [38], etc.) and invoking smart contracts deployed on the blockchain to authenticate the requester’s access token to reduce security issues caused by a large number of IoT devices directly accessing the blockchain network. To reduce the interaction latency with the blockchain, each gateway is deployed as a peer node that holds public data on the chain together.

- Blockchain

The blockchain has two purposes in this system. On the one hand, blockchain acts as a channel of communication between the requester and the owner. The proposed system implements asynchronous communication based on the blockchain network. The communication initiator can initiate a request through a smart contract call, and the receiver can sense the initiator’s request by registering the on-chain callback function, obtaining the corresponding request message body from the chain, and reply it using the same asynchronous communication mechanism.

On the other hand, blockchain acts as a verifier in the ZKP. To confirm the validity of the proof, the owner must construct it off-chain using tamper-evident data from the chain. On-chain verification is then carried out using the pre-uploaded verification key.

- Smart Contract

Based on smart contracts deployed in the chain, we build a series of protocols for interaction between participant entities in the system, which can be divided into three phases: setup phase, access control authorization phase, and token authentication phase. During the setup phase, the smart contract is responsible for the registration of users (requester or owner) and IoT devices, as well as the uploading of verification keys required for ZKP verification. During the access control authorization phase, on the one hand, the smart contract is responsible for facilitating the requester to obtain the user attributes required for authorization matching and initiate an authorization request for access control. On the other hand, the smart contract verifies the ZKP submitted by the owner and returns the required access token to the requester. During the token authentication phase, the smart contract is responsible for providing the gateway to authenticate the legitimacy and validity of the access token.

### 3.2. Sample Access Control

**Figure 2:**
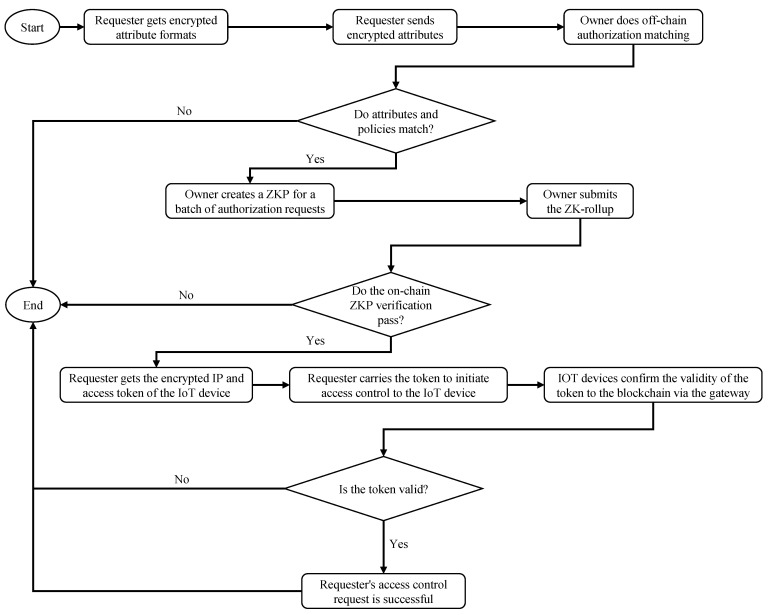
Flow diagram of a sample access control.

A sample access control flow is shown in Figure 2. First of all, the requester asks the owner for the format of the required attributes. Note that the requester and the owner communicate asynchronously via the blockchain. The owner returns the encrypted format to the requester, who prepares the relevant attributes according to the format and transmits them, encrypted, to the owner. The owner matches the attributes submitted by the requester with the predefined policies. The owner creates a ZKP for a batch of authorization requests coming from various requesters. For successfully authorized requests, the owner server will generate the access token, then encrypt tokens and IP addresses of IoT devices using the requester’s public key. The proof, tokens, and IP addresses will be packaged and submitted to the smart contract as the rollup, and the smart contract will verify the ZKP. If the ZKP verification is passed, the requester can obtain the IP and access token through the callback event registered on the blockchain, and apply for access control permission to the IoT device. After the IoT device receives the token from the requester, the token will be verified by the blockchain via the gateway. If the token is legal, the requester officially has access control permission for the target IoT device.

## 4. Access Control Authorization with ZK-Rollups and Protocols

In this section, we will introduce the implementation of access control authorization with ZK-rollups and protocols among participant entities in our proposed system based on the overview of Section 3. Notations that appear in this section are shown in Table 1.

### 4.1. Access Control Authorization with ZK-Rollups

In this subsection, we will go over the design of access control authorization which implements ZK-rollups [24] optimization and ensures the honesty of participants. The zkSNARK framework builds solid proof propositions from arithmetic circuits [22,23]. The arithmetic circuit, which outlines the proof that needs to be constructed, is actually a formal representation of the NP (Non-deterministic Polynomial) statement [34]. By combining this with Section 3, we can conclude that a valid access control authorization procedure meets the following NP statement:

(1) *The owner receives an access control authorization request from a specific requester, as well as the requester attributes for authorization.*
(2) *The owner matches the access attributes with the set policy.*
(3) *The requester obtains the required token to access the IoT device.*

To generate proof from this NP statement, we construct two basic circuits, **“Authorization Matching Circuits”** and **“Access Token Grant Circuits”**.

#### 4.1.1. Authorization Matching Circuits

As shown in Table 2, there are five common access control policies as follows, among which $ID_{req}$, UA, and $ID_{dev}$ need to be generated by the requester side, which we call requester attributes (RA). RA indicates that access control authorization is initiated for the specified IoT device, while EA and AA are generated at the owner server side. Thus, a set of access control attributes can be defined as $A=\left\{RA,EA,AA\right\}=\left\{\left\{ID_{req},ID_{dev},UA\right\},EA,AA\right\}$.

The **“Authorization Matching Circuits”** are responsible for proving the *“the owner matches the access attributes with the set policy”*. An authorization matching process can be described as follows:

(1) Requester uses RA to initiate access control authorization, owner server generates access attributes $A=\left\{RA,EA,AA\right\}=\left\{\alpha_{0},\alpha_{1},\alpha_{2},\cdots ,\alpha_{n}\right\}$ for matching;
(2) Owner uses policy $P=\left\{\rho_{0},\rho_{1},\rho_{2},\cdots ,\rho_{n}\right\}$ to match, if A⊂P, then the authorization access control is legal, otherwise it fails.

Combining the five types of policies shown in Table 2, the proposed system sets the following three attribute-matching patterns.

- **Equivalence Matching.** Determines that $\alpha_{i}$ is exactly equal to $\rho_{i}$, e.g., $ID_{req}$ is consistent with the policy.
- **Range Matching.** Determine $\alpha_{i}$ within the given range of $\rho_{i}$, for example, the timestamp of this access control is Time, the timestamp of last access control is LastTime, and the maximum time interval allowed for consecutive access is GapTime, which needs to satisfy Time−LastTime≤GapTime.
- **Multi-value matching.** Assume $\alpha_{i}=\left\{v_{0},v_{1},v_{2},\cdots ,v_{n}\right\}$, $\rho_{i}=\left\{\theta_{0},\theta_{1},\theta_{2},\cdots ,\theta_{i}\right\}$, multi-value matching needs to judge $\alpha_{i}=\rho_{i}$. For example, the “Access Action” determines whether a requester has read access to all files in a file list.

For **“Equivalence Matching”** and **“Range Matching”**, the general zkSNARK framework comes with based arithmetic circuits to describe such processes, while for **“Multi-value Matching”**, since $\alpha_{i}$ and $\rho_{i}$ need to be considered as a whole, we use the arithmetic circuit of hash operations to describe the matching process, i.e., to determine $Hash(v_{0}\|v_{1}\|v_{2}\left\|\cdots \right\|v_{n})=Hash(\theta_{0}\|\theta_{1}\|\theta_{2}\left\|\cdots \right\|\theta_{n})$.

To guarantee that the owner does use attributes submitted by the requester to do matching, we additionally add an arithmetic circuit of hash operations to determine $Hash\left(RA\right)=Hash(ID_{req}\|ID_{dev}\left\|UA\right)=h_{RA}$. Based on a hash calculation of its submitted requester attributes, the requester generates and appends $h_{RA}$ to the authorization request as a puzzle. The owner must reconstruct this hash value when producing proof to show that it truly makes use of the requester attributes provided by the requester to do authorization matching computation. The existence and correctness of $h_{RA}$ are performed based on the Merkle proof [32], which will be described in Section 4.1.2.

We first need some functions to generate the basic arithmetic circuits and combine these basic components to finally form the target arithmetic circuit, which can represent the NP statement. In combination with the above description, we defined three basic circuit generation functions corresponding to three matching patterns:

- C←CircuitEq(x,y): Construct an arithmetic circuit where *x* and *y* are equal;
- C←CircuitRangeEq(x,y): Construct an arithmetic circuit with *x* in the specified range of *y*. In practice it is a quantitative relationship determined by the matching requirements of access attributes and policies;
- (C,h)←CircuitHash(X): Construct an arithmetic circuit which reveals that $Hash\left(X\right)=Hash(x_{0}\|x_{1}\|x_{2}\|\cdots \|x_{n})$ and output the hash calculation result *h*.

With two additional functions:

- C←NewCircuit(): The arithmetic circuit initialization function.
- A←GenAccAttr(RA): The owner uses $RA.ID_{dev}$ and $RA.ID_{req}$ to query in the owner server to obtain the EA and AA of the corresponding IoT device, and after combining them with RA, the access attribute *A* is generated.

With the above basic circuits and function, we can construct **“Authorization Matching Circuits”** via Algorithm 1.
**Algorithm 1** Build Matching Circuit**Input:** $RA,P,h_{RA}$**Output:** *C*1:C←NewCircuit()2:A←GenAccAttr(RA)3:**for** ($\alpha_{i},\rho_{i}$) in range (A,P) **do**4:    **if** ($\alpha_{i},\rho_{i}$) is **Equivalence Matching then**5:        $C\leftarrow C\cup CircuitEq(\alpha_{i},\rho_{i})$6:    **end if**7:    **if** $\left(\alpha_{i},\rho_{i}\right)$ is **Range Matching then**8:        $C\leftarrow C\cup CircuitRangeEq(\alpha_{i},\rho_{i})$9:    **end if**10:   **if** $\left(\alpha_{i},\rho_{i}\right)$ is **Multi-value matching then**11:        ($C_{\alpha },h_{\alpha })\leftarrow CircuitHash\left(\alpha_{i}\right)$12:        ($C_{\rho },h_{\rho })\leftarrow CircuitHash\left(\rho_{i}\right)$13:        $C^{\prime }\leftarrow CircuitEq\left(h_{\alpha },h_{\rho }\right)$14:        $C\leftarrow C\cup C_{\alpha }\cup C_{\rho }\cup C^{\prime }$15:   **end if**16:    ($C^{\prime },h)\leftarrow CircuitHash\left(RA\right)$17:    $C\leftarrow C\cup C^{\prime }$18:    $C\leftarrow C\cup CircuitEq(h,h_{RA})$19:**end for**

In Algorithm 1, *C* means an arithmetic circuit, $C\leftarrow C_{1}\cup C_{2}$ represents the combination of two circuits. The time and space complexity of Algorithm 1 are related to the size of the (A,P) pair, both being O(N).

#### 4.1.2. Access Token Grant Circuits

**“Access Token Grant Circuits”** are responsible for proving that *“the owner receives an access control authorization request from a specific requester, as well as the requester attributes for authorization”* and *“the requester obtains the required token to access the IoT device”*.

**Figure 3:**
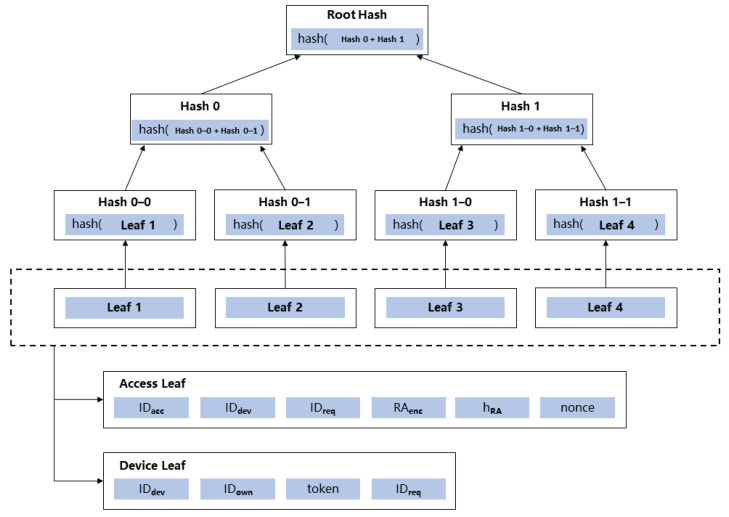
Two kinds of Merkle trees, **“Access Merkle Tree”** and **“Device Merkle Tree”**.

To design arithmetic circuits to implement these two NP statements, we introduce the Merkle proof to confirm: (1) the existence and correctness of the access control authorization request made by the requester and (2) that the IoT device being accessed is in an idle state with no requester for access control. Therefore, we design two different Merkle trees [39], namely, **“Access Merkle Tree”** and **“Device Merkle Tree”**, as shown in Figure 3. These two Merkle trees will be stored permanently in the blockchain to prevent tampering, thus guaranteeing the correctness of the Merkle proof.

Each time a requester initiates an access control authorization, a data structure called “Access” is saved in the public ledger of the blockchain, and its hash value will be added to the **“Access Merkle Tree”** as a leaf. $ID_{acc}$ identifies this access control authorization; $ID_{dev}$ is the ID of the corresponding IoT device; $ID_{req}$ identifies the initiating requester; $RA_{enc}$ is the encrypted form of RA using owner’s public key; $h_{RA}$ is the result Hash(RA) in Section 4.1.1; nonce initiates as 0, which changes to 1 when the access control authorization is completed, through nonce to guarantee that owner cannot repeatedly use the same data structure to construct a proof. By saving these values in the Merkle tree, we can guarantee that (1) the access control authorization is indeed initiated by a requester, an owner cannot arbitrarily grant access control token of the IoT device; (2) $h_{RA}$ exists and is correct, arithmetic circuits of “Access” Merkle proof and Hash(RA) ensure that owner uses requester attributes submitted by the requester to do off-chain authorization matching.

Similarly, the data structure called “Device” identifies the meta info of the IoT device and the current access control status; $ID_{dev}$ and $ID_{own}$ identify the IoT device and its owner. The default value of token and $ID_{req}$ is set to null, which means that no requester is authorized to access this IoT device. As a result, the process of granting an access token can be characterized as first executing a Merkle proof on the token and $ID_{req}$ to ensure that their values are null, after which new token and $ID_{req}$ are assigned, and a new Merkle root hash is calculated. token is generated at the owner server side, and the computation process is shown in Equation (4), salt is the random number generated. The owner needs to inform the requester about the salt, and the requester needs to carry access action AA and salt when initiating formal access control. The gateway repeats the same hash computation process and compares it with the public ledger to determine the correctness of token.

(4) $$token=Hash(ID_{req}\|AA\|salt)$$

In combination with the above description, we have defined a basic circuit:

- C←CircuitVerifyProof(rt,siblings,X): Construct an arithmetic circuit to verify that $Hash\left(X\right)=Hash(x_{0}\|x_{1}\|x_{2}\|\cdots \|x_{n})$ is a leaf node of a Merkle tree with root rt, where siblings are the sibling nodes needed to generate the Merkle proof. As shown in Figure 3, in order to construct the Merkle proof of **Leaf 1**, we need **Hash 0-1**, **Hash 1** as sibling nodes. Then first determines whether hash(**Hash 0-0** + **Hash 0-1**) and **Hash 0** are equal, and then we determine whether hash(**Hash 0** + **Hash 1**) and **Root Hash** are the same. The relevant intermediate arithmetic circuits are generated by CircuitEq and CircuitHash.

With two additional functions:

- siblings←ProofSiblingGeneration(leaves,X): Output the sibling nodes necessary for the Merkle proof, where the hash value of leaf is Hash(X).
- (rt, $leaves_{new})\leftarrow CalMerkleRoot\left(leaves,X^{\prime },X\right)$: leaves means all leaf nodes in the Merkle tree, this function replaces a node in leaves from Hash(X) to $Hash(X^{\prime })$ and output the new Merkle root hash rt, as well as the new Merkle leaf nodes $leaves_{new}$;

With the above basic circuits, we can construct **“Access Token Grant Circuits”** via Algorithms 2 and 3, where dev stands for the “Device” data structure, and acc stands for the “Access” data structure.
**Algorithm 2** Build Device Merkle Circuit**Input:** $dev,{\mathrm{rt}}_{\mathrm{dev}},leaves_{dev},token_{new},ID_{req}^{new}$**Output:** $C,{\mathrm{rt}}_{\mathrm{dev}}^{\mathrm{new}},leaves_{dev}^{new}$1:C←NewCircuit()2:$C\leftarrow C\cup CircuitEq(dev.token,token_{null})$3:$C\leftarrow C\cup CircuitEq(dev.ID_{req},ID_{req}^{null})$4:$siblings_{dev}\leftarrow ProofSiblingGeneration\left(leaves_{dev},dev\right)$5:$C\leftarrow C\cup CircuitVerifyProof({\mathrm{rt}}_{\mathrm{dev}},siblings_{dev},dev)$6:$dev_{new}\leftarrow dev$7:$dev_{new}.token\leftarrow token_{new}$8:$dev_{new}.ID_{req}\leftarrow ID_{req}^{new}$9:$\left({\mathrm{rt}}_{\mathrm{dev}}^{\mathrm{new}},leaves_{dev}^{new}\right)\leftarrow CalMerkleRoot\left(leaves_{dev},dev,dev_{new}\right)$10:$siblings_{dev}^{new}\leftarrow ProofSiblingGeneration\left(leaves_{dev}^{new},dev_{new}\right)$11:$C\leftarrow C\cup CircuitVerifyProof({\mathrm{rt}}_{\mathrm{dev}}^{\mathrm{new}},siblings_{dev}^{new},dev_{new})$

**Algorithm 3** Build Access Merkle Circuit**Input:** $acc,{\mathrm{rt}}_{\mathrm{acc}},leaves_{acc}$**Output:** $C,{\mathrm{rt}}_{\mathrm{acc}}^{\mathrm{new}},leaves_{acc}^{new}$
1:

C←NewCircuit()

2:

C←C∪CircuitEq(acc.nonce,0)

3:

$siblings_{acc}\leftarrow ProofSiblingGeneration\left(leaves_{acc},acc\right)$

4:

$C\leftarrow C\cup CircuitVerifyProof({\mathrm{rt}}_{\mathrm{acc}},siblings_{acc},acc)$

5:

$acc_{new}\leftarrow acc$

6:

$acc_{new}.nonce\leftarrow 1$

7:

C←C∪CircuitEq(acc.nonce,1)

8:

$\left({\mathrm{rt}}_{\mathrm{acc}}^{\mathrm{new}},leaves_{acc}^{new}\right)\leftarrow CalMerkleRoot\left(leaves_{acc},acc,acc_{new}\right)$

9:

$siblings_{acc}^{new}\leftarrow ProofSiblingGeneration\left(leaves_{acc}^{new},acc\right)$

10:

$C\leftarrow C\cup CircuitVerifyProof({\mathrm{rt}}_{\mathrm{acc}}^{\mathrm{new}},siblings_{acc}^{new},acc_{new})$




The time and space complexity analysis of Algorithms 2 and 3 is performed. Suppose the size of leaves is *N*. For the function ProofSiblingGeneration, in order to obtain the sibling nodes, a Merkle tree needs to be constructed through the parameter leaves, and the time complexity required for the construction is O(N). CalMerkleRoot and CircuitVerify require a sequential hash computation from the leaf nodes to the root node with a time complexity related to the tree height of O(logN). Therefore, the time complexity of the above algorithms is O(N+logN+logN)=O(N). Furthermore, the space complexity of the above two algorithms is related to the space complexity of the constructed Merkle trees, which are both O(N).

In practice, we choose to keep the height of the Merkle tree within a certain range for four reasons:

- As the tree height increases, the time overhead of Algorithms 2 and 3 execution will be concentrated on ProofSiblingGeneration, which will slow down the authorization overall, as we will show in Section 5.2 in conjunction with experimental data.
- ProofSiblingGeneration also needs to bring Merkle leaves into memory for generating proof, considering an IoT environment with a large number of devices and access authorization requests, a tree with a height of 30 can verify about 1 billion leaf nodes, and a leaf hash of 256 bits will bring a memory overhead of $2^{30}$× 256 bit = 32 GB.
- The article [40] shows that the height of the Merkle tree only affects how many leaves can be verified with the Merkle proof, and has no effect on the security of proof, which is mainly determined by the hash function.
- The owner needs to query Merkle leaves from the public ledger; taller trees mean heavier transmission delay.

For the **“Device Merkle Tree”**, since a single owner has limited IoT devices, for every owner, we generate a tree with its identifier. For **“Access Merkle Tree”**, since the number of authorization requests from the requester may grow infinitely, we choose to have the smart contract reconstruct a Merkle tree and return the identifier of the tree to the users whenever $M_{acc}$ leaf nodes are generated. $M_{acc}$ is usually set to $2^{10}$ to make authorization execute efficiently.

#### 4.1.3. Zero-Knowledge Rollup Circuits

We take into account the characteristics of huge instantaneous access control requests in IoT environments while implementing zero-knowledge access control authorization, combining this with the characteristic of centralized off-chain computation at the owner-server side in the proposed system. As a result, we use the ZK-rollup [24] concept and decide to create a ZK proof for a batch of access control authorization requests before submitting it to the smart contract for ZK proof verification. This will increase the throughput of the owner server side, decrease the time cost of authorization verification, and lower on-chain computation overhead.

**Figure 4:**
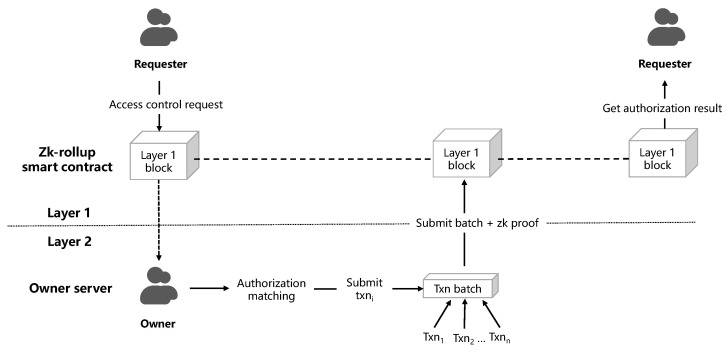
Procedure of the ZK-rollup in the proposed system.

Figure 4 depicts the ZK-rollup procedure in its entirety. The owner server simultaneously receives authorization requests from various requesters. Following off-chain authorization matching of access attributes and policy, each authorization request is treated as a transaction. When combined with other authorization requests, these transactions form the rollup of access control authorization. Then, the ZK proof is created using the generated rollup and sent for proof verification to the blockchain smart contract.

We can construct final ZK-rollup circuits at the owner side via Algorithm 4.

The time and space complexity analysis of Algorithm 4 is performed. Assuming that $leaves_{acc}$ and $leaves_{dev}$ have size $N_{leaves}$, policies *P* and requester attributes RA have size $N_{match}$, and $batchSize=N_{bacth}$. When batchSize=0, ProofSiblingGeneration function in Algorithms 2 and 3 is called for the first time, at which time a Merkle tree is constructed, so the time complexity of ProofSiblingGeneration is $O(N_{leaves})$. When batchSize>0, ProofSiblingGeneration can obtain the corresponding sibling nodes based on the same Merkle tree, so the time complexity of the ProofSiblingGeneration function is related to the tree height, which is $O(logN_{leaves})$. Combined with the time complexity analysis of Section 4.1.2, the time complexity of Algorithms 2 and 3 is $O(N_{leaves})$ when batchSize=0, and $O(logN_{leaves})$ when batchSize>0. Therefore, the time complexity of Algorithm 4 is as follows:

(5) $$O(N_{leaves}+N_{batch}\times \left(logN_{leaves}+N_{match}\right))$$

**Algorithm 4** Build ZK-Rollup Circuit**Input:** $List_{acc},List_{dev},{\mathrm{rt}}_{\mathrm{acc}},{\mathrm{rt}}_{\mathrm{dev}},leaves_{acc},leaves_{dev},List_{token},List_{ID_{req}},List_{P}$**Output:** $C,{\mathrm{rt}}_{\mathrm{acc}},{\mathrm{rt}}_{\mathrm{dev}}$1:C←NewCircuit(),i←02:${\mathrm{rt}}_{\mathrm{acc}}^{\mathrm{before}}\leftarrow {\mathrm{rt}}_{\mathrm{acc}}$3:${\mathrm{rt}}_{\mathrm{dev}}^{\mathrm{before}}\leftarrow {\mathrm{rt}}_{\mathrm{dev}}$4:**while** i<batchSize **do**5:    $\left(C_{acc},{\mathrm{rt}}_{\mathrm{acc}}^{\mathrm{new}},leaves_{acc}^{new}\right)\leftarrow BuildAccessMerkleCircuit\left(List_{acc}\left[i\right],{\mathrm{rt}}_{\mathrm{acc}},leaves_{acc}\right)$6:    $\left(C_{dev},{\mathrm{rt}}_{\mathrm{dev}}^{\mathrm{new}},leaves_{dev}^{new}\right)\leftarrow BuildDeviceMerkleCircuit\left(List_{dev}\left[i\right],{\mathrm{rt}}_{\mathrm{dev}},leaves_{dev},List_{token}\left[i\right],List_{ID_{req}}\left[i\right]\right)$7:    $C_{m}\leftarrow BuildMatchCir\left(List_{acc}\left[i\right].RA,List_{P}\left[i\right],List_{acc}\left[i\right].h_{RA}\right)$8:    $C\leftarrow C_{acc}\cup C_{dev}\cup C_{m}$9:    $C\leftarrow C\cup CircuitEq(List_{acc}\left[i\right].ID_{req},List_{dev}\left[i\right].ID_{req})$10:    $C\leftarrow C\cup CircuitEq(List_{acc}\left[i\right].ID_{dev},List_{dev}\left[i\right].ID_{dev})$11:    **if** i>0 **then**12:        $C\leftarrow C\cup CircuitEq({\mathrm{rt}}_{\mathrm{acc}},{\mathrm{rt}}_{\mathrm{acc}}^{\mathrm{before}})$13:        $C\leftarrow C\cup CircuitEq({\mathrm{rt}}_{\mathrm{dev}},{\mathrm{rt}}_{\mathrm{dev}}^{\mathrm{before}})$14:    **end if**15:    ${\mathrm{rt}}_{\mathrm{acc}}^{\mathrm{before}}\leftarrow {\mathrm{rt}}_{\mathrm{acc}}$16:    ${\mathrm{rt}}_{\mathrm{acc}}\leftarrow {\mathrm{rt}}_{\mathrm{acc}}^{\mathrm{new}}$17:    ${\mathrm{rt}}_{\mathrm{dev}}^{\mathrm{before}}\leftarrow {\mathrm{rt}}_{\mathrm{dev}}$18:    ${\mathrm{rt}}_{\mathrm{dev}}\leftarrow {\mathrm{rt}}_{\mathrm{acc}}^{\mathrm{dev}}$19:    $leaves_{acc}\leftarrow leaves_{acc}^{new}$20:    $leaves_{dev}\leftarrow leaves_{dev}^{new}$21:    i←i+122:**end while**

Because Algorithm 4 needs to construct two Merkle trees and perform $N_{batch}$ authorization matching during its execution, the space complexity is:

(6) $$O(N_{leaves}+N_{batch}\times N_{match})$$

In Algorithm 4, $List_{acc}$ where $RA_{enc}$ is decrypted into RA and $List_{dev}$ is a list of data structure “Access” and “Device”; $List_{token}$ is a list of access tokens for requesters to access to IoT devices, which is also generated by the owner; the identifiers in $List_{ID_{req}}$ identify requesters, meaning requester’s access permission; $List_{P}$ is policies stored in a local database of owner server. The size of the lists above is batchSize, which is equal to the size of transactions. In lines 9–11, the generated circuits will verify the intermediate Merkle root hash to ensure the continuity of the token grant process of the rollup.

It is important to note that Algorithm 4 shows the authorization process for a successful authorization matching, and the generation algorithm for circuits that fail to match only removes the arithmetic logic associated with the **“Device Merkle Tree”**. It does not need to consider the process of the token grant, the token and $ID_{req}$ maintain default null value, since lines 6, 9–10, 17–18, and 20 can be removed.

### 4.2. Protocols of System

In this subsection, we will explain the protocols of the proposed system, which are divided into three phases, the setup phase, the access control authorization phase, and the token authorization phase.

To verify the caller’s identity, all protocols—aside from **“Register User”** and **“Token Authentication”**—must be verified for signatures on the smart contract. The caller (requester or owner) uses $SIG=Sign\left(Hash\left(X\right),SK_{sig}\right)=Sign(Hash(x_{0}\|x_{1}\left\|\cdots \right\|x_{n}),SK_{sig})$ to generate the signature, and the smart contract uses $\left\{0,1\right\}\leftarrow SigVerify\left(SIG,Hash\left(X\right),PK_{sig}\right)$ for signature verification, where *X* is the list of parameters of the smart contract, and $<PK_{sig},SK_{sig}>$, is the public-private key pair used for the signature. In the above procedure, the signature of the caller is generated based on the hash of the parameter list; in the SigVerify function, the smart contract first uses $PK_{sig}$ to obtain the plaintext of SIG and compares it with Hash(X), if they are the same, the signature verification is successful.

#### 4.2.1. Setup Phase

**Protocol 1. Register User**

- **Goal:** register users in the proposed system, which may be owners or requesters
- **Smart contract parameters:** $\left\{PK_{enc},PK_{sig},type\right\}$

**Steps:**

(1) Requester $U_{req}$ or owner $U_{own}$, generate locally the public-private key pair for encryption $<PK_{enc},SK_{enc}>=KeyGen_{enc}\left(r\right)$ and the public-private key pair for signature $<PK_{sig},SK_{sig}>=KeyGen_{sig}\left(r\right)$, *r* is the randomness.
(2) Upload $\left\{PK_{enc},PK_{sig},type\right\}$ to the smart contract, type is the user type.
(3) Generate globally unique user identifier $ID_{user}$ by smart contract and store user information $U=\left\{ID_{user},PK_{enc},PK_{sig}\right\}$ in public ledger.

**Protocol 2. Register Device**

- **Goal:** the owner registers the owned IoT device with the blockchain network
- **Smart contract parameters:** $\left\{ID_{dev},ID_{own}\right\}$

**Steps:**

(1) Owner $U_{own}$ generates locally the identifier $ID_{dev}$ of the owned device and the public-private key pair for encryption $<PK_{enc}^{dev},SK_{enc}^{dev}>=KeyGen_{enc}\left(r\right)$.
(2) Submit $\left\{ID_{dev},ID_{own},PK_{enc}^{dev}\right\}$ to the smart contract.
(3) The smart contract stores the information of the IoT device $D=\{ID_{dev},ID_{own},token_{null},$$ID_{req}^{null},PK_{enc}^{dev}\}$ to the public ledger, where $token_{null}$ and $ID_{req}^{null}$ represent the default token and $ID_{req}$ which shows that no requesters can use a token to access to the IoT device, and adds a leaf node to the **“Device Merkle Tree”** marked as $ID_{own}$.
(4) Register a callback event with the owner server so that the owner is aware that a requester has initiated access control authorization for the IoT device.

**Protocol 3. Verification Key Setup**

- **Goal:** upload verification key of ZK proof to smart contract
- **Smart contract parameters:** $\left\{ID_{own},vk\right\}$

**Steps:**

(1) Owner $U_{own}$ generates *C* with Algorithm 4, input parameters ${\mathrm{rt}}_{\mathrm{dev}},{\mathrm{rt}}_{\mathrm{acc}}$, $leaves_{acc}$, $leaves_{dev}$ are queried from the public ledger, the rest of the parameters can be generated through a batch of past authorization requests.
(2) Use *C* to generate the proving key pk and verification key vk from the Equation (1), as well as the R1CS ccs required by *w* and *x* in Equation (2), store pk and ccs in the local server.
(3) $\left\{ID_{own},vk\right\}$ is uploaded to the smart contract, which generates a globally unique vk identifier $ID_{vk}$ and stores $V=\left\{ID_{vk},ID_{own},vk\right\}$ to the public ledger.

#### 4.2.2. Access Control Authorization Phase

**Figure 5:**
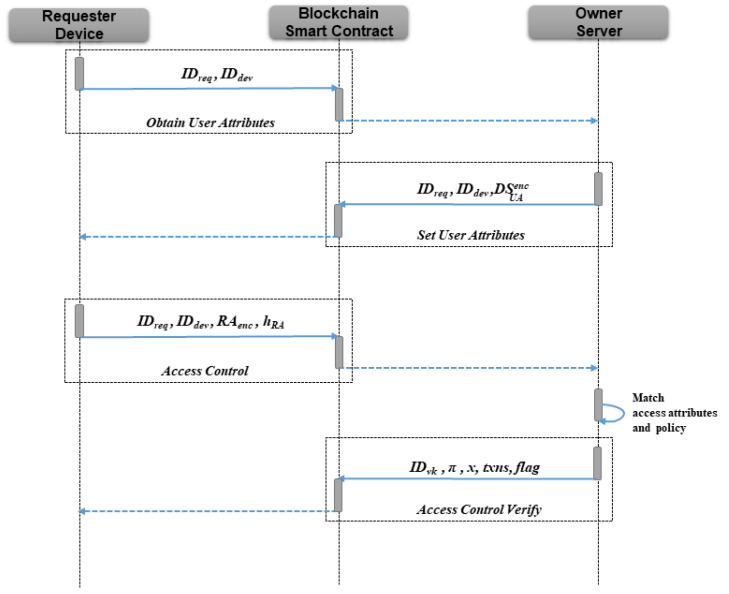
Procedure of the access control authorization phase.

The access control authorization phase procedure is described in Figure 5.

**Protocol 4. Obtain User Attributes**

- **Goal:** let the requester request user attributes (UA) from owner for access control authorization
- **Smart contract parameters:** $\left\{ID_{req},ID_{dev}\right\}$

**Steps:**

(1) Requester uploads $\left\{ID_{req},ID_{dev}\right\}$ to the smart contract to obtain the needed user attributes for a specific IoT device.
(2) The smart contract will store the requester attributes information in the public ledger $RA_{req}=\left\{ID_{req},ID_{dev}\right\}$.
(3) Register a callback event with the requester device so that the requester detects the owner’s modification of $RA_{req}$, then triggers the callback event registered by the owner in **Register Device**.

**Protocol 5. Set User Attributes**

- **Goal:** let the owner inform the requester of the required user attributes format for authorization
- **Smart contract parameters:** $\left\{ID_{req},ID_{dev},DS_{UA}^{enc}\right\}$

**Steps:**

(1) The owner senses the request of getting user attributes format through the callback event and stores the policy *P* required in the server.
(2) Prepare the data structure $DS_{UA}=\left\{\eta_{0},\eta_{1},\cdots ,\eta_{n}\right\}$, where $\eta_{i}=\left(name,type\right)$, indicating the name and type (string/int/unsigned int array) of a single user attribute.
(3) Obtain the $PK_{enc}^{req}$ of the corresponding requester from the public ledger and use the encryption function to obtain $DS_{UA}^{enc}=ENC\left(DS_{UA},PK_{enc}^{req}\right)$.
(4) Call the smart contract to add $DS_{UA}^{enc}$ to the corresponding $RA_{req}=RA_{req}\cup DS_{UA}^{enc}$, and then trigger the callback event registered by **Get User Attributes**.

**Protocol 6. Access Control**

- **Goal:** requester formally initiates access control authorization
- **Smart contract parameters:** $\left\{ID_{req},ID_{dev},RA_{enc},h_{RA}\right\}$

**Steps:**

(1) Requester detects that the owner has set the user attributes format required for authorization via the callback event.
(2) Decrypt $DS_{UA}=DEC\left(DS_{UA}^{enc},SK_{enc}^{req}\right)$ using the private key, and then generate the RA required for authorization locally.
(3) Obtain the $PK_{enc}^{own}$ of the corresponding owner from the public ledger, and use the encryption function to obtain $RA_{enc}=ENC\left(RA,PK_{enc}^{own}\right)$ and compute $h_{RA}=Hash\left(RA\right)$.
(4) Call the smart contract.
(5) Smart contract generates the identifier $ID_{acc}$ for this access control authorization in the public ledger and stores $ACC=\left\{ID_{acc},ID_{req},ID_{dev},RA_{enc},h_{RA},nonce=0,ID_{Merkle}^{acc}\right\}$ to the public ledger.
(6) Add a leaf node to the **“Access Merkle Tree”**, the identifier of tree generated by smart contract is $ID_{Merkle}^{acc}$.
(7) Trigger the callback event for the owner registered in **“Register Device”**, informing the owner that a requester has initiated authorization.

**Protocol 7. Access Control Verify**

- **Goal:** owner integrates authorization requests into a batch of transactions, constructs the zero-knowledge proof, and submits them to smart contracts for verification, the authorization matching result of transactions should be all successful or failed.
- **Smart contract parameters:** $\left\{ID_{vk},\pi ,x,txns,flag\right\}$

**Steps:**

(1) Following the receipt of a predetermined number of access control authorizations, the owner begins building the circuit *C* using Algorithm 4, where the public ledger is queried for the input parameters ${\mathrm{rt}}_{\mathrm{dev}},{\mathrm{rt}}_{\mathrm{acc}}$, $leaves_{acc},leaves_{dev}$, and the remaining parameters are built using the authorization requests received.
(2) In *C*, all $ID_{acc}$ in the batch, initial ${\mathrm{rt}}_{\mathrm{dev}}$ and ${\mathrm{rt}}_{\mathrm{acc}}$ before access control authorization are set to *x* that can be made public, then read ccs and pk previously saved locally to construct the proof π using Equation (2);
(3) Owner uploads $\left\{ID_{vk},\pi ,x,txns,flag\right\}$ to the smart contract, where flag is the authorization result type of π (success/failure), and $txns=\left\{txn_{0},txn_{1},\cdots ,txn_{n}\right\},txn_{i}=\left\{ID_{acc},token,time_{token},IP_{dev}^{enc},salt_{enc},AA_{enc}\right\}$. $time_{token}$ represents the duration of the requester to access the device. IP, salt, and AA are encrypted using the requester’s encryption public key. Furthermore, token is generated using Equation (4).
(4) The smart contract verifies that all $ID_{acc}$ in $List_{ID_{acc}}$ exist in *x* and obtains ${\mathrm{rt}}_{\mathrm{acc}}$, ${\mathrm{rt}}_{\mathrm{dev}}$ from *x* to compare with the values in the current public ledger, if they are equal, the corresponding vk is obtained by $ID_{vk}$ and executes verification using Equation (3), otherwise the smart contract will return.
(5) After executing verification, if the verification value is 1, the smart contract assigns a value of 1 to the nonce and records π in ACC corresponding to $ID_{acc}$, then assign token and $ID_{req}$ to the corresponding “Device” if flag is true. If the verification value is 0, the smart contract will reject the batch of authorization verification.

#### 4.2.3. Token Authentication Phase

**Protocol 8. Token Authentication**

- **Goal:** requester uses the token to access the IoT device

**Steps:**

(1) Requester and IoT device establish the session key $K_{rd}$ based on key exchange algorithm (RSA [41]/DH [42]/ECDHE [43]) using their respective public keys. The communication process between the requester and the IoT device will then be encrypted via $K_{rd}$.
(2) Requester initiates a new access control command, encrypts salt and AA using $K_{rd}$, which is then sent to the IoT device.
(3) The IoT device decrypts to obtain salt and AA, also obtains token in the current public ledger via the gateway, and uses Equation (4) to check the correctness of token. If token is legitimate, the IoT device generates a random number $N_{d}$ to send to the requester.
(4) Requester encrypts $N_{d}$ with $K_{rd}$ and sends it to the IoT device. The IoT device decrypts $N_{d}$ and compares it with the previous value to achieve strong authentication of the requester.
(5) To make sure that $N_{d}$ is fresh, the requester must repeat steps (2)–(4) when a new access control command is initiated.

## 5. Evaluation

### 5.1. Implementation Details

We develop a prototype of the proposed system on Hyperledger Fabric 2.2 [44], using fabric-go-sdk for blockchain smart contract calls, implemented in Golang 1.19 programming language, and the zkSNARK framework used is gnark [45]. The hash arithmetic logic of the circuit and the protocol uniformly use MIMC [46] as the hash function. The cryptographic algorithm of ecc [47] is used in the protocol to encrypt sensitive data, and EdDSA [48] is used as the signature algorithm. The key exchange algorithm uses the ECDHE [43].

The prototype is built on a server with Intel(R) Xeon(R) Platinum 8269 CY CPU @ 2.50 GHz, 16G RAM, and Ubuntu 20.04 OS. Based on the architecture shown in the system overview, we create three virtual machines (VMware Workstation 16) on the server as the owner server, requester device, and gateway, then deploy fabric peer nodes on each of them. The number of processors per virtual machine is set to 2 and the memory is set to 4 GB. A Raspberry Pi (CPU: 1.5 GHz ARM Cortex-A, Memory: 4G) is also connected to the virtual machine that acts as a gateway.

### 5.2. Experiment Results

**Figure 6:**
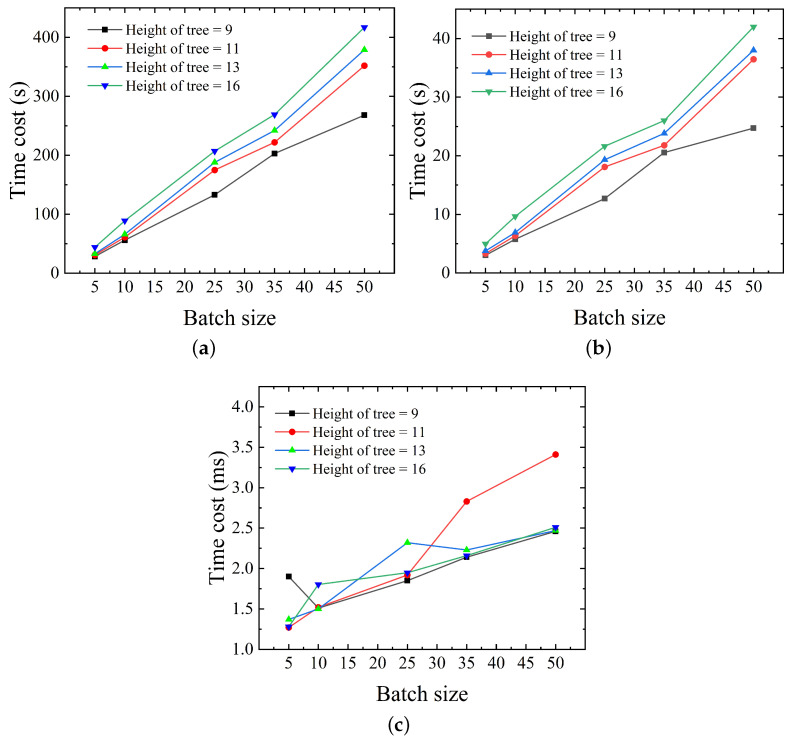
Time cost of functions related to ZK proof. (**a**) Time cost (s) of KeyGen. (**b**) Time cost (s) of Prove. (**c**) Time cost (ms) of Verify.

**Figure 7:**
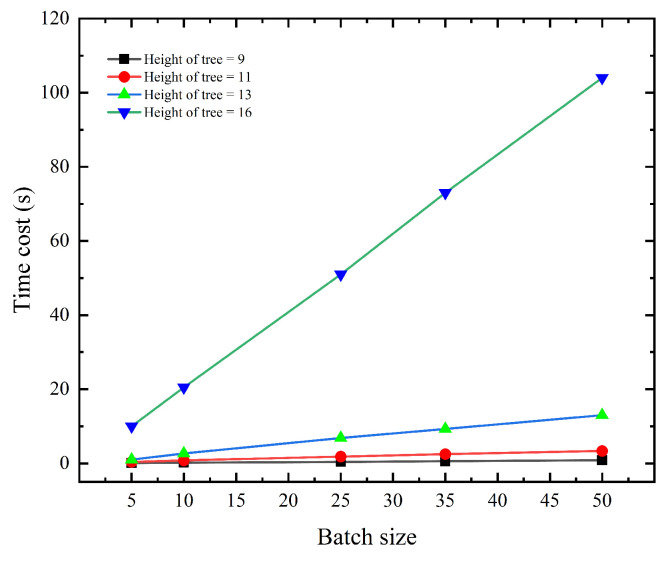
Time cost (s) of Algorithm 4, Build ZK-Rollup Circuit.

In the proposed system, we implement eight kinds of protocols. The time cost of protocols 1, 2, 4, 5, 6, 8 can be steadily controlled in less than 5 s. The running time of protocols 3, 7 is related to three functions KenGen,Prove,Verify required to perform zero-knowledge proof [22] and Algorithm 4, BuildRollupCircuit. In practice, the time cost of ZK-related functions and Algorithm 4 may be affected by the batch size and height of Merkle trees. To find the effect of the above two factors on the time cost, in the experiment we set the height of **“Access Merkle Tree”** and **“Device Merkle Tree”** to 9, 11, 13, 16, as shown in Figure 6 and Figure 7.

The time needed for KenGen is the longest of the three ZK-related functions, as illustrated in Figure 6a. When the batch size is 12 or smaller, the total time consumed can be managed within 100 s, but if the batch size surpasses 50, the total time consumed exceeds 200 s. In general, the time used grows linearly with the increase in batch size. Fortunately, KenGen is a preprocessing function that generates the pk and vk required for authorization, and this part is off-chain computation, which is performed in protocol **“Verification Key Setup”** in advance. The owner can pre-generate various arithmetic circuit types on the server and obtain the corresponding pk and vk, preventing the KeyGen function from slowing down the whole authorization procedure.

The time consumption of function Prove, which is used for access control authorization, is depicted in Figure 6b. The time overhead of Prove also increases linearly with batch size, although it is still possible to keep the total time overhead under 50 s. By choosing the right batch size, we can in fact reduce this portion of the overall time overhead.

Figure 6c illustrates the amount of time required by the procedure for Verify, an on-chain computation carried out by the smart contract. As shown in Figure 6c, we can observe that the time spent in this portion can be consistent at the single-digit millisecond level and is less influenced by the batch size and tree height. As a consequence, the very small zero-knowledge verification time allows the proposed system’s on-chain computation overhead to remain stable, and when the authorization result is published to the chain, it can be immediately accepted by all peer nodes.

Figure 7 illustrates the amount of time required by the procedure for Algorithm 7. The image demonstrates that the time overhead of this component is minimal when Height = 9, 11, 13 but drastically increases when Height = 16. This is because the proposed system only stores the leaf nodes, necessitating a recalculation of the intermediate values from the leaf nodes to the root node when the function ProofSiblingGeneration in Section 4.1.2 is used to construct the root hash rt. This is the reason why we keep the maximum number of “Access” generated consecutively to $M_{acc}=2^{10}$ in Section 4.1.2.

The memory use of the produced pk and vk is displayed in Table 3 and Table 4, where the input arithmetic circuit is generated by Algorithm 4. The memory footprint of pk grows from tens to hundreds of MB as the tree height and batch size rise, but the memory footprint of vk merely grows as the batch size does. pk takes up more space compared to vk, but pk is only used to generate the proof π off the chain, while vk, which needs to participate in Verify function calls on the chain, only takes up KB-level space in the public ledger, which does not significantly increase the storage overhead of peer nodes. The size of π generated by Prove is constant at 1024 bits, which also does not increase the storage overhead on the chain.

Next, we will compare the performance of existing systems with respect to the access authorization process. Systems by Maesa et al. [21], Hu et al. [20], and ours are presented. The system by Maesa et al. only considers the authorization matching process, and the system by Hu et al. only considers the access token grant process. Our system includes both processes. Operation (modular or monolithic) of Evaluate SMART POLICY in the system by Maesa et al. generates the zero-knowledge proof of authorization matching off the chain and submits the proof to the chain for verification; $tx_{dele}$ in the system by Hu et al. completes the zero-knowledge token grant. We divide the entire access authorization process into two parts, the off-chain computation, and on-chain computation.

**Figure 8:**
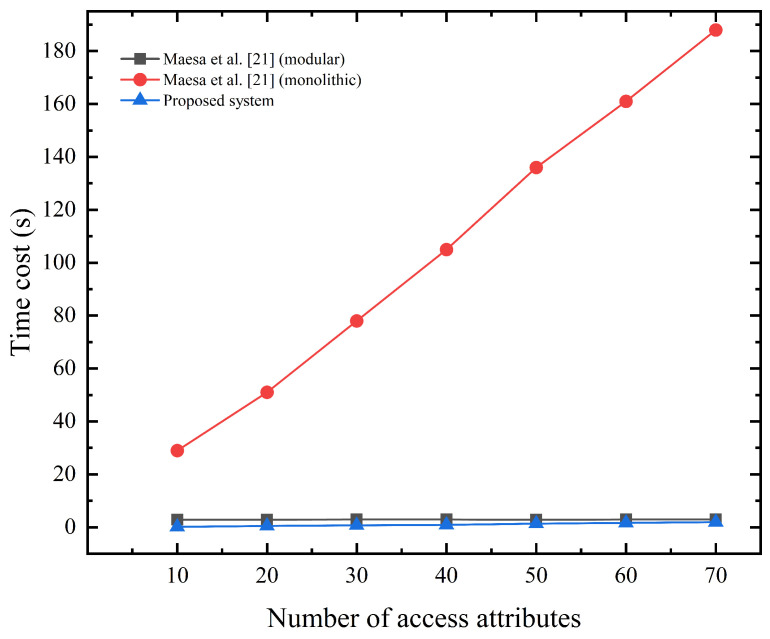
Zero-knowledge proof generation time (s) off the chain of Maesa et al.’s system [21] and ours.

The off-chain computation mainly involves the generation of the zero-knowledge proof. We first compare the system of Maesa et al. with the proposed system. As shown in Figure 8, the time cost of generating the zero-knowledge proof for our system and the modular approach stays consistent as the number of access attributes rises, but the monolithic approach’s time cost keeps rising. To ensure that the generated proofs contain the knowledge related to the accessed attributes, both systems add hash puzzles similar to Section 4.1.1 to the proofs. For the modular approach and our system, the strategy of generating an overall hash puzzle for all attributes is adopted, so the proof generation time remains stable; while for the monolithic approach, each attribute in the attribute set has to generate a specific hash puzzle, so the time overhead continues to rise.

Both Hu et al.’s system and ours construct arithmetic circuits containing the Merkle proof. The system by Hu et al. concentrates all tokens in a Merkle tree with a height of 32, which leads to two problems. (1) Proof generation time becomes longer: the average proof generation time for each request named $tx_{dele}$ is 28.9 s; while for ours, as shown in Figure 6b, when the batch size is 5, our system can control the proof generation time in about 5 s, and the average generation time corresponding to each request can be controlled in 1 s. (2) Merkle proof generation time becomes longer: we reduce the height of the Merkle tree in the Hu et al. system to 20, which still takes about 61.5 s to generate the Merkle proof. Section 4.1.2 mentions that the proposed system chooses to regenerate a **“Access Merkle Tree”** every $M_{acc}=2^{10}$ authorization requests, and controls the height of the tree in this way.

**Figure 9:**
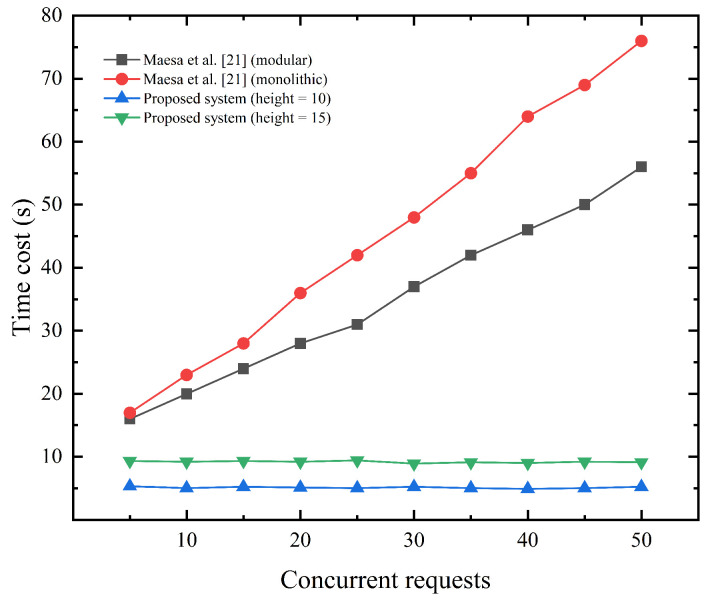
On-chain computation time cost (s) for system of Maesa et al. [21] and ours in the low-traffic environment.

The on-chain computation mainly includes smart contract execution, node consensus agreement, and network communication. Smart contracts contain ZKP verification and system-varied business logic, such as registered device verification, access token granting, etc. Network communication includes two different ways, between users and blockchain nodes or between nodes. Since the work of Hu et al. does not mention the relevant smart contract implementation, we mainly compare the performance of Maesa et al.’s system with ours for on-chain computation in a low-traffic environment. Maesa et al.’s system is implemented based on Ethereum, and we set the average block time of this system to 12 s, which is consistent with the practical application environment https://ycharts.com/indicators/ethereum_average_block_time (accesed on 13 March 2023). In addition, we control the number of concurrent requests in the experimental environment at the same time to 5–50 for simulating a low-traffic environment. As shown in Figure 9, as the number of concurrent requests increases, the time for on-chain computation continues to increase in the system by Maesa et al. regardless of the modular approach or the monolithic approach. For the proposed system, we set the height of the associated Merkle tree to the common 10 or 15, and the time cost can remain stable in the low-traffic environment.

**Figure 10:**
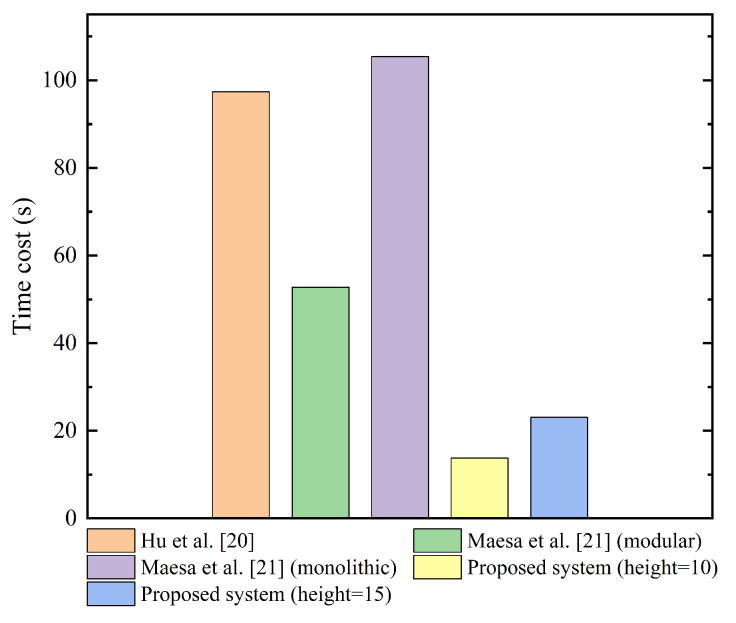
Time cost (s) of access control authorization process in the low-traffic environment, where the number of concurrent requests is set to 50.

As shown in Figure 10, we combine the on-chain and off-chain computation and compare the overall access authorization process performance for different systems. For the system by Hu et al., we still shrink the height of the Merkle tree to 20, and choose to deploy a ZKP-verified contract on the Hyperledger Fabric. We set the number of concurrent requests in the experimental environment to 50. Combining the previous discussion and experimental results of Figure 10, the proposed system has a lower time cost of the overall access authorization process in the low-traffic environment compared to the other two systems.

**Figure 11:**
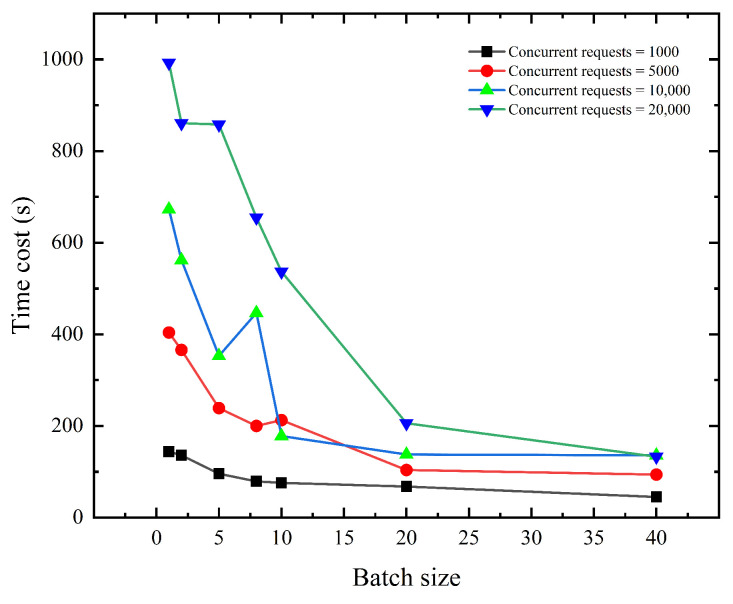
Time cost (s) of the protocol **“Access Control Verify”** in the environment with many concurrent requests.

To further demonstrate the optimization of our system for high-traffic IoT environments, we design an experimental environment with a large number of concurrent requests. Figure 11 displays the total amount of time spent by the protocol **“Access Control Verify”** when the **“Device Merkle Tree”** and **“Access Merkle Tree”** heights are 10 and the experimental environment simulates the situation where there are 1000, 5000, 10,000, 20,000 concurrent smart contract requests in the blockchain network, 40 total access control requests that need to be authorized, and every batch size is set to 1, 2, 5, 8, 10, 20, 40. According to Figure 11, the time required by the protocol in the same scenario decreases exponentially as the batch size increases. When comparing different scenarios of existing concurrent requests, we can see that time consumption varies significantly when the batch size is 1, even reaching 993 s when there are 20,000 concurrent requests. The difference in time consumption between various scenarios gradually lowers as the batch size grows. It can be observed that with a batch size of 40, the time cost can be reduced to 133 s, which is an 86% reduction compared to the case of a batch size of 1.

The following is an analysis of the above experimental results of Figure 11. For zero-knowledge access control authorization in **“Access Control Verify”**, the off-chain computation needs to consider the time overhead of Algorithm 4 Build ZK-Rollup Circuit and generate a proof, and this part of the overhead can be optimized by controlling the tree height and batch size. For on-chain computation, we need to consider the transmission delay of each invocation to the smart contract and the time for each peer node to reach an agreement using the consensus algorithm. Therefore, based on the ZK-rollup method, aggregating the access control authorization in fewer batches can reduce the number of smart contract calls and reduce transmission latency in congested network environments on the one hand; on the other hand, since the Verify time on the chain is stable at the single-digit millisecond level as shown in Figure 6c, fewer batches mean fewer on-chain consensus algorithm calls. The experimental results demonstrate that the ZK-rollup-based optimization can significantly reduce the time consumed by access control authorization in high-traffic environments, thus making the proposed system more suitable for IoT environments with a large number of devices and instantaneous accesses.

## 6. Security Analysis

In this section, we will provide a security analysis to demonstrate the system’s ability to guarantee user honesty.

### 6.1. Potential Attack Modes and Defenses

- Attack 1: a malicious requester wants to obtain the privileges of a device by forging the requester attributes in an existing authorization request. On the one hand, π is generated based on zkSNARK, which does not expose any information about access attributes and policies, and on the other hand, user attributes are encrypted using the requester’s private key, which cannot be restored by the attacker via $RA_{enc}$. Furthermore, the format $DS_{UA}$ of the user attribute UA is not exposed, because $DS_{UA}$ is also encrypted during the transmission.
- Attack 2: a malicious requester initiates a replay attack, reusing ACC in the public ledger to initiate access control authorization. If the nonce is 1, the owner server will not perform authorization matching after receiving the request. If the nonce is found to be 0, although the malicious requester obtains the authorization result through Protocol 7, it cannot obtain salt, AA, and the IP of the device through decryption. The malicious requester can neither initiate access control to the IoT device nor obtain any knowledge about token.
- Attack 3: a malicious owner receives an access authorization request from a requester and has the corresponding policy in its server, but the owner chooses to forge non-existent requester attributes to perform the authorization matching. $Hash(List_{acc}\left[i\right].RA)$$=List_{acc}\left[i\right].h_{RA}$ in the arithmetic circuit to ensure that the owner can only use the submitted requester attributes for matching, and proof of **“Access Merkle Tree”** ensures that $h_{RA}$ exists and any forged requester attributes do not pass the Merkle proof test.
- Attack 4: a malicious owner wishes to bypass the rules of authorization matching and arbitrarily grant access permission of an IoT device to a requester. This system ensures that this behavior does not occur in three ways: first, the malicious owner cannot build a non-existent proof of **“Access Merkle Tree”**, which ensures that an owner can only grant the token through an authorization request initiated by a requester; second, nonce, if equal to 1, ensures that requests that have completed authorization matching are not reused; third, nonce if equal to 0, with line 9 of Algorithm 4 ensures the correspondence between the requester and the authorization request, the malicious owner cannot use the request initiated by a specific requester to grant token to another requester.
- Attack 5: the attacker aims to take control of an IoT device by replaying previous requests during the **“Token Authentication”** phase. To prevent the attacker from obtaining the most recent $N_{d}$, the AA and salt are encrypted using the symmetric secret key $K_{rd}$. Moreover, the freshness of $N_{d}$ assures that an attacker cannot obtain access control by using $N_{d}$’s previous value.

### 6.2. Formal Security Verification Using AVISPA

We test the security of our suggested protocols in Section 4.2 against an adversary using the widely used AVISPA [49], which is a formal security software verification tool. In AVISPA, a security protocol is tested to see if it is safe or unsafe using the “High-Level Protocol Specification Language (HLPSL)”.

**Figure 12:**
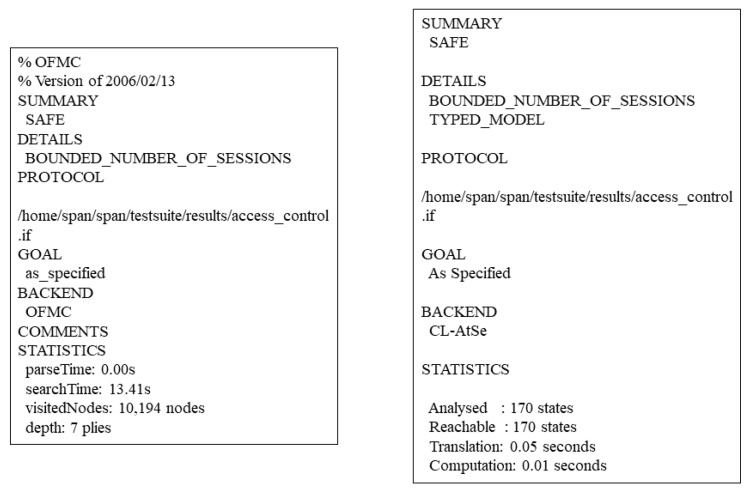
Results of AVISPA’s analysis of the **“Access Control Authorization”** phase.

**Figure 13:**
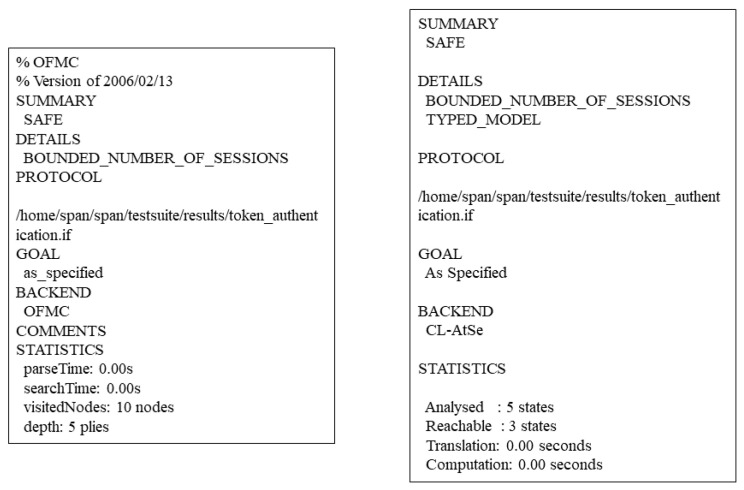
Results of AVISPA’s analysis of the **“Token Authentication”** phase.

We analyze the protocols of the **“Access Control Authorization”** phase and **“Token Authentication”** phase using AVISPA. For the **“Setup”** phase, we consider that the information generated in this phase is publicly available in the blockchain network, so there is no relevant analysis. We use different authentication mechanisms for the two phases. For the **“Access Control Authorization”** phase, we want the system to complete the authorization faster with less communication in this phase, so we use a weak authentication mechanism and the wrequest function in HLPSL for authentication. In this phase, even if an attacker of the Dolev-Yao intruder model [50] hijacks the intermediate message, the attacker cannot obtain the information related to the requester attributes and token because of the asymmetric encryption algorithm of the proposed system. For protocol of the **“Token Authentication”** phase, we use a strong authentication mechanism, hoping that an attacker cannot masquerade as a requester and gain access control permission without knowing any information about token, so we use the request function in HLPSL for authentication. The HLPSL code is available in the URL https://github.com/SheenLin/ZKRollups-AC (accessed on 13 March 2023). In the protocol analysis, we use OFMC and CL-AtSe as backend, and the results of the protocol security analysis are shown in Figure 12 and Figure 13.

## 7. Conclusions

### 7.1. Research Summary

The blockchain-based AC system with ZKP [22,23,29,30] is currently a popular area of study [19,20,21]. However, current solutions cannot be adaptive to high-traffic IoT environments, also cannot fully guarantee user honesty. We propose a system based on the above considerations. The proposed system is based on the ZK-rollups [24] concept, where specialized Merkle trees [39] and a hash puzzle are designed to construct the zero-knowledge proof. The authorization matching and token grant processes from different authorization requests are encoded into arithmetic circuits. The generated proofs conform to the NP statement for access control authorization and effectively prevent malicious users from initiating attacks on the system, ensuring the honesty of participants and the non-disclosure of sensitive data such as access attributes and policies. We also introduce the communication protocols among participant entities in the system, which are based on a series of arithmetic circuit generation algorithms to effectively improve performance in high-traffic environments and the security of the system.

Through a solid experimental design, this work also illustrates how the proposed system can be used in an IoT scenario. A reasonable design of batch size and Merkle tree height can stabilize the time required for the protocols. The produced pk solely consumes off-chain memory, and vk does not significantly increase the on-chain memory occupation, according to experimental data. We split the entire access control authorization process into two processes: off-chain and on-chain computation, and we compare how long each process takes on existing systems and ours. We also show that the proposed system has the least access control authorization time cost in low-traffic IoT environments by integrating the two processes. Further, we demonstrate that in high-traffic environments, the proposed system can cut the authorization time overhead by 86% based on the ZK-rollups optimization. In the security analysis, we construct five different attack modes and show how our system can protect user honesty by demonstrating that it can resist these malicious behaviors. In addition, we verify the security of the system protocols through the AVISPA tool [49].

### 7.2. Future Work

Currently, our proposed system still has some limitations, but it is moving in the direction in which our future work will go.

1. Each participant entity in the proposed system must maintain a public-private key pair since the conventional asymmetric encryption key management technique is used. However, in an IoT environment with high traffic and many participating entities, such a key management method is undoubtedly very complicated. To lessen the difficulties of key management on the one hand and provide fine-grained access control to encrypted data on the other, we intend to add attribute-based encryption [51] in future work.
2. In practice, we found that using Algorithm 1 to generate authorization matching circuits is strongly related to the specific business. Despite the fact that we have established three matching patterns, users still need to construct several authorization matching circuits beforehand to meet various business needs. To maximize the effectiveness of authorization matching circuit generation, we want to further improve Algorithm 1 and provide relevant template configuration techniques in future work.
3. Currently, the proposed system takes into account that in a high-traffic IoT environment, the owner’s side may receive a large number of access authorization requests instantaneously, so it adopts the idea of ZK-rollups to design the Protocol **“Access Control Verify”**. However, the requester also needs to send a large number of access authorization requests at the same time to obtain the privileges of an IoT device. Therefore, in future work, we plan to use similar batch optimization on the requester side to improve the performance of the related protocols.

## Figures and Tables

**Table 1:** Notations List.

Notation	Description
*A*	The access attribute set used for authorization matching
$\alpha_{i}$	The *i*th access attribute in *A*
*P*	The policy set used for authorization matching
$\rho_{i}$	The *i*th policy in *P*
*C*	The arithmetic circuit
Hash	Hash function
*h*	The hash calculation result
dev	The “Device”data structure in the public ledger
acc	The “Access” data structure in the public ledger
leaves	A list of leaf nodes in the Merkle tree
siblings	Sibling nodes needed to generate the Merkle proof of a leaf
token	The token required for the requester to perform access control on a device
salt	Randomness used to generated token
$PK_{enc},PK_{sig}$	Public key for encryption or signature generation
$SK_{enc},SK_{sig}$	Private key for encryption or signature generation
$KeyGen_{enc},KeyGen_{sig}$	The generation function of <PK,SK> pair
ENC,DEC	Encryption and decryption functions
Sign	The signature generation function
SigVerify	The signature verification function
$DS_{UA}$	Data structure describing the composition form of UA
pk,vk	Proving key and verification key of zkSNARK
*x*	Input message of zkSNARK
*w*	Witness of zkSNARK
txns	The transactions of ZK-rollups
π	Generated non-interactive proof of zkSNARK
$K_{rd}$	Symmetric session key for requester and IoT device

**Table 2:** Common Access Control Policies.

Policy Type	Description
Requester Identity ($ID_{req}$)	Used to specify the requester’s identity
IoT Device Identity ($ID_{dev}$)	Used to specify the IoT device’s identity
User Attributes (UA)	Characteristics used to describe a requester, such as role, age, department, and job
Environmental Attributes (EA)	Used to describe the environmental characteristics of the IoT device when access control occurs, such as time, temperature, etc.
Access Action (AA)	Used to specify the IoT device operations that the requester is permitted to perform, such as read, write, execute, etc.

**Table 3:** Size (MB) of proving key, where the input arithmetic circuit is generated by Algorithm 4 Build ZK-Rollup Circuit.

Height of Tree	Batch Size = 5	Batch Size = 10	Batch Size = 25	Batch Size = 35	Batch Size = 50
9	30	60	143	220	287
11	33	66	189	239	378
13	35	71	202	258	405
16	47	95	223	287	446

**Table 4:** Size (KB) of verification key, where the input arithmetic circuit is generated by Algorithm 4 Build ZK-Rollup Circuit.

Batch Size = 5	Batch Size = 10	Batch Size = 25	Batch Size = 35	Batch Size = 50
0.94	1.56	3.44	4.69	6.56

## Data Availability

The data used to support the findings of this study are available from the corresponding author upon request.
